# The Probiotic Yeast, Milmed, Promotes Autophagy and Antioxidant Pathways in BV-2 Microglia Cells and *C. elegans*

**DOI:** 10.3390/antiox14040393

**Published:** 2025-03-27

**Authors:** Federica Armeli, Beatrice Mengoni, Emily Schifano, Thomas Lenz, Trevor Archer, Daniela Uccelletti, Rita Businaro

**Affiliations:** 1 Department of Medico-Surgical Sciences and Biotechnologies, Sapienza University of Rome, 04100 Latina, Italy; federica.armeli@uniroma1.it (F.A.); beatrice.mengoni@uniroma1.it (B.M.); 2 Department of Human Sciences, European University of Rome, 00163 Rome, Italy; 3 Department of Biology and Biotechnologies “C. Darwin”, Sapienza University of Rome, 00185 Rome, Italy; emily.schifano@uniroma1.it (E.S.); daniela.uccelletti@uniroma1.it (D.U.); 4 Milmed Unico AB, 11139 Stockholm, Sweden; thomas.lenz@milmed.se (T.L.); trevorcsarcher49@gmail.com (T.A.); 5 NMR-Based Metabolomics Laboratory (NMLab), Sapienza University of Rome, 00185 Rome, Italy

**Keywords:** yeast, microglia, neuroinflammation, autophagy, oxidative stress, NRF2, *Caenorhabditis elegans*, probiotic, longevity, ROS

## Abstract

**Background:** Autophagy, a catabolic process essential for maintaining cellular homeostasis, declines with age and unhealthy lifestyles, contributing to neurodegenerative diseases. Probiotics, including Milmed yeast, have demonstrated anti-inflammatory and antioxidant properties. This study evaluated the activity of Milmed on BV-2 microglial cells in vitro and in the in vivo model of *Caenorhabditis elegans* (*C. elegans*) in restoring autophagic processes. **Methods:** BV-2 microglial cells were incubated with *S. cerevisiae* (Milmed treated yeast or untreated yeast) and then stimulated with lipopolysaccharide (LPS). mRNAs of the autophagic factors and antioxidant enzymes were assessed by qPCR; mTOR and NRF2 were evaluated by ELISA. pNRF2 compared with cytosolic NRF2 was evaluated by immunofluorescence. The longevity, body size, and reactive oxygen species (ROS) levels of *C. elegans* were measured by fluorescence microscopy. **Results:** Treatment with Milmed YPD cultured yeast or the dried powder obtained from it promoted autophagic flux, as shown by the increased expression of the *Beclin-1*, *ATG7*, *LC3*, and *p62* mRNAs and the inhibition of mTOR, as evaluated by ELISA. It also enhanced the antioxidant response by increasing the expression of *NRF2*, *SOD1*, and *GPX*; moreover, pNRF2 expression compared with cytosolic NRF2 expression was enhanced, as shown by immunofluorescence. Milmed dietary supplementation prolonged the survival of *C. elegans* and reduced the age-related ROS accumulation without changing the expression of *gst-4.* The pro-longevity effect was found to be dependent on SKN-1/Nrf2 activation, as shown by the absence of benefit in *skn-1* mutants. **Conclusions:** Milmed yeast demonstrates significant pro-autophagy and antioxidant activity with significant pro-longevity effects in *C. elegans*, thereby extending the lifespan and improving stress resistance, which, together with the previously demonstrated anti-inflammatory activity, highlights its role as a highly effective probiotic for its beneficial health effects. Activation of the SKN-1/NRF2 pathway and the modulation of autophagy support the therapeutic potential of Milmed in neuroprotection and healthy aging.

## 1. Introduction

Autophagy, as an evolution-restricted, lysosome-dependent catabolic process present in all eukaryotic cells, plays an essential maintenance role in the homeostatic response to stress and other extrinsic and intrinsic strains placed upon an organism, promoting cell survival and degrading damaged and dysfunctional intracellular organelles [1,2,3]. Nevertheless, autophagic efficacy deteriorates with advancing age, sedentary lifestyle and unrestricted, nonselective nutrition leading to chronic disease states such as obesity, sarcopenia, type 2 diabetes, inflammaging, and oxidative stress [4,5,6]. Neuronal survival, homeostatic processes, and the regulation of nutrient uptake and energy balance necessitate an ongoing autophagy [7,8]. The links between autophagy and aging processes have been established in longevity models of *Caenorhabditis elegans* whereby neurodegenerative propensities and a decline in health parameters and longevity are associated with compromised autophagy with concomitant expressions of inflammaging and oxidative stress [9].

Probiotic agents, living microorganisms with minimal or the absence of adverse reactions, have been applied for the treatment of a wide range of disease/disorder states as well as offering generalized health benefits and protective advantages [10,11,12]. It has been shown that probiotics induce health-enhancing effects by engaging a variety of mechanisms including lowering the intestinal pH, decreasing colonization and invasion by pathogenic organisms, and modifying the host immune response. In this context, it has been implied that probiotics may redress existing bacterial-fungal gut microbiome actions, thereby reducing the inflammatory and/or toxic propensities [13,14,15]. These agents have been shown to induce preventative/protective actions in several animal models of tissue disorder including intestinal damage, intestinal injury gastroenteritis, cardiovascular tissue damage, and improved renal function following injury [16,17,18,19]. The probiotic, *Lactiplantibacillus plantarum* NJAU-01, was found to produce ameliorative effects upon D-galactose-induced hepatic oxidative stress through the induction of the hepatic total antioxidant capacity and antioxidant enzyme activities involved in superoxide dismutase, glutathione peroxidase, and catalase [20]. In a systematic review presenting the beneficial effects of probiotics through the promotion of autophagy and a comparison of the intestinal cell lines and/or tissue with other types of cell lines and tissue, Nemati et al. (2021) postulated that autophagy effectively describes a viable mechanism to describe the health benefits of probiotic actions over a range of tissue substrates [21]. An effective yeast probiotic, a *Saccharomyces cerevisiae* strain isolated from a traditional Korean fermentation initiator, was found to reduce the levels of interleukin-1β and interferon-γ. Furthermore, the probiotic yeast, *Saccharomyces boulardii*, was administered to transgenic APP/PS1 mice expressing neuroinflammation arising from activated microglia and the toll-like receptor pathway; this treatment alleviated dysbiosis and improved cognitive impairment [15]. Yeasts have contributed to the understanding of fundamental aspects of lifespan regulation including the roles of nutrient response, global protein translation rates and quality, DNA damage, oxidative stress, mitochondrial function and dysfunction as well as autophagy [22].

It was previously found that the treated yeast, Milmed, exerted both neuroprotective and neurorestorative effects both functionally and neurochemically in a lower, repeated dose, progressive administration of -methyl-4-phenyl-1,2,3,6-tetrahydropyridine (MPTP), a selective dopamine neurotoxin, as a laboratory animal model of Parkinson’s disease [23,24]. Yeast cells that have undergone treatment with millimeter-wavelength electromagnetic waves (range: 1 GHz to 300 GHz), so-called Milmed, were found to exert a high level of anti-allergy efficacy among suffering patients [25]. Furthermore, patients presenting with irritable bowel syndrome and symptoms of inflammatory bowel disease (IBS-IBD) reported fewer symptoms following treatment compared with patients who had a placebo (untreated yeast preparation) and expressed fewer symptoms compared with their pre-treatment report [26,27,28]. Armeli et al. (2022) demonstrated that Milmed yeast, derived from *S. cerevisiae* exposed to millimeter wavelengths (as developed by Golant 1994 and Golant et al. 1994 [28,29]), induced a shift in LPS-M1-polarized microglia toward an anti-inflammatory phenotype. This transition was evidenced morphologically by the restoration of the resting microglial phenotype, as shown by the reduced inducible nitric oxide synthase (iNOS) and the reduced mRNA levels of IL-1β, IL-6, and TNF-α [27].

Furthermore, Milmed stimulates the secretion of interleukin-10 and the expression of arginase-1, which provides cell markers of M2 anti-inflammatory polarized cells. Among racehorses in training presenting with physiological health problems, mainly ‘common-cold’ and inflammation symptoms remain a constant setback. In two studies, I and II, the time to reduce the pulse to 130 and pulse rate after 15-minutes were estimated both before and after the Milmed treatment, and the levels of improvement on a scale of 1–10 as well as judgements of vigor, general health, and performance following several weeks of Milmed administration were assessed [26,30].

In both studies, it was indicated that the Milmed-treated racehorses presenting poor health showed an improved physiological health responses, underlying the utility of several weeks of Milmed yeast treatment for the alleviation of mainly respiratory conditions among racehorses under the relatively stressful training and racing season. Remarkably, no evidence of toxicity, neither in vitro nor in vivo, has ever been obtained with the Milmed yeast [31].

Taking into account the several health-rendering observations attributed to the Milmed yeast, it was contended that the probiotic may promote the advancement of autophagic processes among cell cultures. Thus, in all the observations, liposaccharide (LPS) was applied to induce cellular damage/toxicity in order to assess the Milmed propensities for anti-inflammatory, autophagic, and antioxidant properties: the direct anti-inflammatory effect through the augmentation of arginine-1 following the exposure of BV-2 microglia to pro-inflammatory LPS was assessed. The promotion of autophagy was measured through Beclin-1, a protein that is encoded by the BECN1 gene in humans, which promotes the induction of autophagy, and ATG7, which recruits proteins related to autophagy. The formation of the phagophore begins, which enlarges to become an autophagosome factor as well as P62, a multifunctional ubiquitinated binding protein that is involved in the signaling pathways of many cell life activities including autophagy and has an impact upon cell survival [32,33,34,35]. NRF2, a basic leucine zipper (bZip) transcription factor with a Cap’n’collar (CNC) structure, accumulates in the cytoplasm and then shifts into the nucleus to activate the transcription of downstream genes that code for antioxidant enzymes, which protect cells from oxidative damage. It facilitates the transcription of the downstream antioxidant genes in disc cells by binding to antioxidant response elements (AREs) in promoter regions [36,37,38]. Evaluation and mRNA analysis of the factors involved in autophagic flux (RT-PCR) and enzyme linked immunosorbent assay (ELISA) were performed in BV-2 cells [32].

Given the well-established role of oxidative stress in aging and neurodegenerative processes, model organisms such as *Caenorhabditis elegans* provide an invaluable system for studying the molecular mechanisms underlying stress resistance and longevity [39]. *C. elegans* is widely recognized for its genetic tractability, short lifespan, and highly conserved stress response pathways, making it a powerful tool for investigating the effects of dietary and pharmacological interventions on oxidative homeostasis and lifespan regulation [40]. In this study, we utilized nematodes to evaluate the potential antioxidant and pro-longevity effects of Milmed in vivo. The transgenic *gst-4*::GFP worm strain, a well-established reporter strain for assessing the activation of the SKN-1/NRF2 pathway, which is a central regulator of cellular redox balance, was employed. Additionally, we analyzed the lifespan of *skn-1* mutant strains to determine the contribution of these stress-responsive pathways to the observed effects. Reactive oxygen species (ROS) levels were measured at different time points to assess whether yeast supplementation influenced the oxidative damage accumulation over time. By integrating different approaches in the BV-2 microglia cells and *C. elegans* in vivo model, this study aimed to elucidate the potential mechanisms through which Milmed yeast may promote cellular stress resistance and longevity, providing new insights into the interplay between dietary factors, oxidative stress, and autophagy.

## 2. Materials and Methods

### 2.1. Cell Culture and Treatment

The BV-2 murine microglial cell line, kindly provided by Dr. Mangino, Sapienza University of Rome (Italy), was cultured in Dulbecco’s modified Eagle’s medium (DMEM, Euroclone, Pero, MI, Italy) supplemented with 10% fetal bovine serum (FBS; Sigma-Aldrich, St. Louis, MO, USA) and 1% penicillin-streptomycin (Sigma-Aldrich, St. Louis, MO, USA) at 37 °C in a humidified incubator under 5% CO_2_ until they reached 90% confluence. Cells were seeded in 6-well plates (cell density of 10^6^ cells/well) and incubated at 37° C with 10^3^ Milmed yeast, a commercial probiotic (Alnozine™) obtained from *Saccharomyces cerevisiae* after exposure to millimeter electromagnetic wavelengths (range: 1 GHz to 300 GHz) [28,29]. The dried yeast preparation was produced by the established yeast manufacturer, Jästbolaget AB, Sollentuna, Sweden. The yeast was treated in a suspension by applying an apparatus that generated electromagnetic millimeter waves, followed by the intensification of the suspension yeast manufactured, and then drying it to a powder through an air-drying technique. Milmed was administered to the microglia cultures either in liquid form (Milmed) or in the liquid form of dried powder regrown in YPD medium (Milmed YPD), or as a powder directly added to the cultures (dried Milmed). Prior to each treatment, a viability assay was performed to count the live yeast cells/each treatment. Microglia cultures were also incubated with the common *S. cerevisiae* strain (untreated yeast) as a control. After 45 min, the BV-2 cells were added with lipopolysaccharide (LPS, strain 0111:B4, Sigma-Aldrich, St. Louis, MO, USA) at a concentration of 1 ng/mL and compared with the control.

### 2.2. Real-Time Quantitative PRC Analysis

The total RNA was extracted from both the BV2 control and treated cells using the miRNeasy Micro Kit (Qiagen, Hilden, Germany) and quantified by a NanoDrop One/OneC (Thermo Fisher Scientific, Waltham, MA, USA). The cDNA was synthesized using a high-throughput reverse transcription kit. Quantitative real-time PCR (qPCR) was performed for each sample in triplicate on an Applied Biosystems 7900HT fast real-time PCR system (Applied Biosystem, Cheshire, UK) using Power SYBR^®^ Green PCR Master Mix (Applied Biosystem, Cheshire, UK). Primers for real-time PCR amplification were designed through the UCSC Genome Browser https://genome.cse.ucsc.edu/ (accessed on 1 November 2022); University of California, Santa Cruz, CA, USA) (Table 1). Analysis of the real-time PCR data was performed using the comparative threshold cycle (CT) method. The target quantity, normalized against the endogenous β-actin reference primer (∆CT) and against the untreated control calibrator (∆∆CT), was calculated by the 2^−∆∆CT^ equation.

### 2.3. ELISA Assay

To distinguish the LC3-II form from the LC3-I form, the LC3-II antigen was analyzed by the ELISA assay. Cell lysates were prepared and analyzed according to the Autophagy ELISA Kit (LC3-II Quantitation, Cell Biolabs, INC, San Diego, CA, USA). To quantify mTOR kinase and NRF2, cell lysates were prepared and analyzed according to mTOR KLM1703 GENLISA^TM^ Mouse Mammalian Target of Rapamicyn ELISA 96T (KRISHGEN BioSystems Mumbai, India), and according to the Autophagy ELISA Kit LC3-II Quantitation CBA-5116 (Cell Biolabs, San Diego, CA, USA). The absorbance (450 nm) was determined by a Varioskan™ LUX Multimode Microplate Reader Thermo Scientific (Waltham, MA, USA).

### 2.4. Immunofluorescence

A total of 30,000 BV2 cells/well of the chamber slides were plated in 200 μL of 10% DMEM FBS and stimulated with 10^3^ Milmed, dried Milmed, and untreated yeast in the presence and absence of LPS 1 ng/mL for 24 h. After 3 washes in PBS, cells were fixed in 4% paraformaldehyde for 30′ RT. After 3 washes, TritonX-100 0.1% × 5′ was added. Two washes in PBS were performed, and glycine 0.1 M was added for 20′ RT. After 2 further washes in PBS, the cells were incubated with Primary Antibody 1:100 in PBS 01% BSA O.N. 4 °C for anti-NRF2 (NRF2 (A-10), Santa Cruz Biotechnology, Dallas, TX, USA) and anti-pNRF2 (NRF2 phospho Ser40, GeneTex, Irvine, CA, USA). After 3 washes in PBS, the cells were incubated with the fluoresceinated secondary antibody (Rb CF488-a goat anti-Rb Ig(H+L) 20012, Biotium) for 30′ RT (1:100 in PBS) in the dark. After washing off the excess, the slides were sealed with Vectashield^®^ Antifade Mounting Medium with DAPI (Newark, NJ, USA). Qualitative images were acquired using a confocal ZEISS Ism 980 with ZEISS Zen Microscopy Software 3.6 (Carl Zeiss Inc., Ober Kochen, Germany). Fluorescence intensity was quantified with ImageJ software, version 1.48.

### 2.5. C. elegans Strains and Lifespan Analysis

The *C. elegans* strains used in this study were wild-type N2, CL2166 (dvIs19 [(pAF15)gst-4p::GFP::NLS] III), and QV225 *skn-1* (zj15). Synchronized nematodes were prepared as described in Schifano et al. (2022) [41] on NGM plates spread with 60 µL of *S. cerevisiae* BY4741, untreated yeast, or Milmed at a final concentration of 10^8^ cells/mL. Yeast strains were cultured in YPD broth and incubated at 28 °C overnight aerobically. Lifespan analysis was conducted at 16 °C, with worms transferred daily to fresh plates seeded with newly prepared yeast lawns. Worms were scored dead when they no longer responded to gentle touch with a platinum wire. A minimum of 80 nematodes per condition was used in each experiment. All lifespan assays were performed in triplicate.

### 2.6. Body Size Analysis

Synchronized N2 worms were incubated at 16 °C on NGM plates seeded with Milmed or *S. cerevisiae* BY4741, prepared as previously described, allowing the embryos to hatch and develop. For body length measurements, animals were photographed at 1- and 8-days post-hatching using a ZEISS Axiovert 25 microscope connected to an Axiocam 208 color camera. The worm body length was determined using ZEISS ZEN Microscopy Software 2011 and compared with the BY4741-fed worms. At least 30 nematodes were analyzed per dataset, with a minimum of three independent experiments conducted.

### 2.7. Fluorescence Analysis of C. elegans Transgenic Strains

At day 4 of adulthood, synchronized *gst-4*::GFP transgenic worms fed with Milmed or untreated yeast from embryo hatching were anesthetized with sodium azide (20 mmol L^−1^) (Sigma-Aldrich, St. Louis, MO, USA) and observed using a ZEISS Axiovert 25 microscope, as described in Schifano et al. (2022) [41]. Each experiment was repeated three times, with 15 worms per group analyzed in each replicate. Images were acquired with an exposure time of 0.2 s, and the fluorescence intensity was quantified using ImageJ software. Scale bars were added using Zeiss ZEN Microscopy Software 2011.

### 2.8. Evaluation of Reactive Oxygen Species (ROS) Levels

The ROS levels in 1- and 10-day-old adult worms, fed with Milmed or untreated yeast from embryo hatching, were measured using the fluorescent probe 2′,7′-dichlorofluorescein diacetate (H_2_DCFDA), according to Ficociello et al. (2023) [42]. Briefly, worms were collected in triplicate in a 96-well microplate and washed with M9 buffer. The H_2_DCFDA probe (Sigma-Aldrich, Milan, Italy) was added to each sample to a final concentration of 50 μM. After 1 h of incubation in the dark at 20 °C, fluorescence was measured using a multiple reader (Promega, Glomax Multidetection System, Madison, WI, USA) at excitation/emission wavelengths of 485/520 nm. Fluorescence was detected in whole worms because the H_2_DCFDA probe is cell-permeable and undergoes hydrolysis by intracellular esterases, followed by oxidation by reactive oxygen species (ROS), leading to fluorescence. It should be taken into account that in worm homogenates, the fluorescence signal can be significantly reduced or absent because the esterases might be inactivated or lost during homogenization, and the ROS levels may be lower or more rapidly degraded in the disrupted cellular environment. Several works have reported the measurements of *C. elegans* ROS in entire animals [43,44].

### 2.9. Statistical Analyses

Data were expressed as the mean values ± standard deviations (SD) or mean values ± SEM from at least three independent experiments. Statistical analyses were performed using the unpaired Student’s *t* test (GraphPad Software Inc., San Diego, CA, USA). All results were considered statistically significant with *p* < 0.05. For the *C. elegans* experiments, the statistical significance was performed by the Student’s t-test or one-way ANOVA analysis coupled with a Bonferroni post-test (GraphPad Prism 9.0 software, GraphPad Software Inc., La Jolla, CA, USA). Differences with *p* values < 0.05 were considered significant and were indicated as follows: * *p* < 0.05, ** *p* < 0.01, and *** *p* < 0.001. Experiments were performed at least in triplicate. Data were presented as the mean ± SD. For the fluorescence images, the mean fluorescence intensity was analyzed using ImageJ software, measuring the ratio of pixels per area of the worm.

## 3. Results

### 3.1. MILMED Restores Autophagic Processes in LPS-Treated BV2 Cells

**Figure 1:**
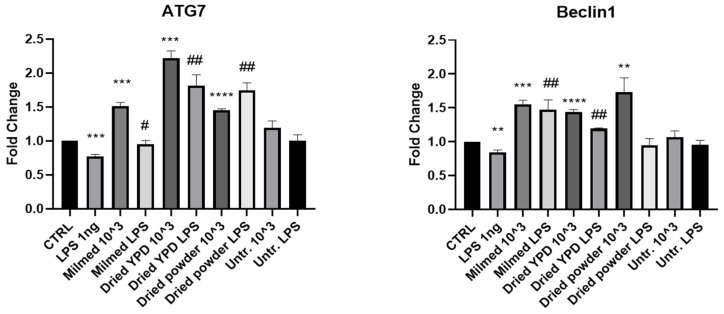
mRNA expression of *Beclin-1* and *ATG7* in BV-2 cells after yeast pre-treatment and LPS stimulation. mRNA expressions of *Beclin-1* and *ATG7* were evaluated in BV-2 cells after pre-treatment with Milmed or untreated yeast for 45 min and incubated with LPS 1 ng/mL for 4 h by qRT-PCR. Data are shown as the mean ± SD from three independent experiments performed in triplicate. Expression profiles were determined using the 2^−ΔΔCT^ method. vs. CTRL ** *p* < 0.01, *** *p* < 0.001, **** *p* < 0.0001. vs. LPS # *p* < 0.05, ## *p* < 0.01.

As known, the autophagic process is composed of several steps that lead to the degradation of misfolded proteins and the elimination of damaged organelles. We evaluated the expression of the genes that promote autophagosome formation, namely ATG7, which is involved in the formation of the pre-autophagosomal structures; the Beclin-1 factor, which intervenes in the initial stages of nucleation and forms a complex that facilitates the recruitment of other ATG proteins; and LC3 and LC3II markers of autophagosome vescicles. Our results showed that cells incubated with Milmed increased the expression of *Beclin-1* mRNA by 50% also in the presence of LPS, which by itself induced a 20% decrement of *Beclin-1* mRNA expression. An analogous increase in *Beclin-1* mRNA was observed after the addition of Milmed dried powder. Cells supplemented with the untreated yeast showed no difference compared with the control cells. Milmed and Milmed dried powder increased the expression of *ATG7* by 50% compared with the control cells but Milmed dried powder regrown in YPD showed a 100% increase in *ATG7* mRNA (Figure 1).

**Figure 2:**
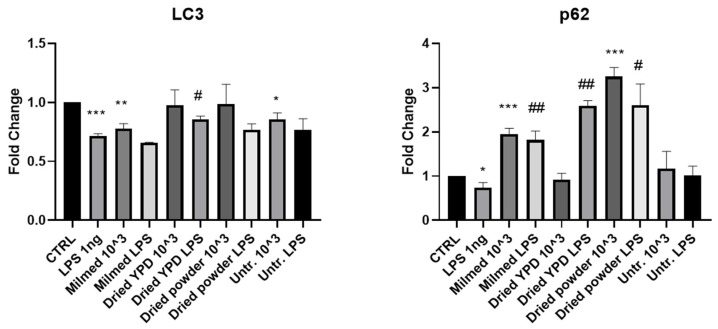
mRNA expression of *LC3* and *p62* in BV-2 cells after yeast pre-treatment and LPS stimulation. mRNA expressions of *LC3* and *p62* were evaluated in BV-2 cells after pre-treatment with Milmed or untreated yeast for 45 min and incubated with LPS 1 ng/mL for 4 h by qRT-PCR. Data are shown as the mean ± SD from three independent experiments performed in triplicate. Expression profiles were determined using the 2^−ΔΔCT^ method. vs. CTRL * *p* < 0.05, ** *p* < 0.01, *** *p* < 0.001; vs. LPS # *p* < 0.05, ## *p* < 0.01.

The interaction between p62 and LC3 represents the specific and necessary signal to direct cytosolic elements to degradation. Analysis of *LC3* and *p62* mRNA expression, as shown in Figure 2, demonstrated that the presence of the LPS inflammatory stimulus significantly reduced the expression of these two key factors of autophagic flux compared with the CTRL group. After treatment with Milmed, both *LC3* and *p62* mRNA expression increased in the BV2 cells. Milmed treatment also increased the *p62* mRNA expression in the presence of LPS. The addition of dried yeast regrown in YPD significantly increased the expression of *LC3* and *p62* in the presence of the inflammatory stimulus. Addition of the Milmed dried powder directly to the BV2 cell culture increased the *p62* expression both in the absence and presence of LPS. The untreated yeast decreased *LC3* expression compared with the CTRL group and had no effect on *p62* expression.

**Figure 3:**
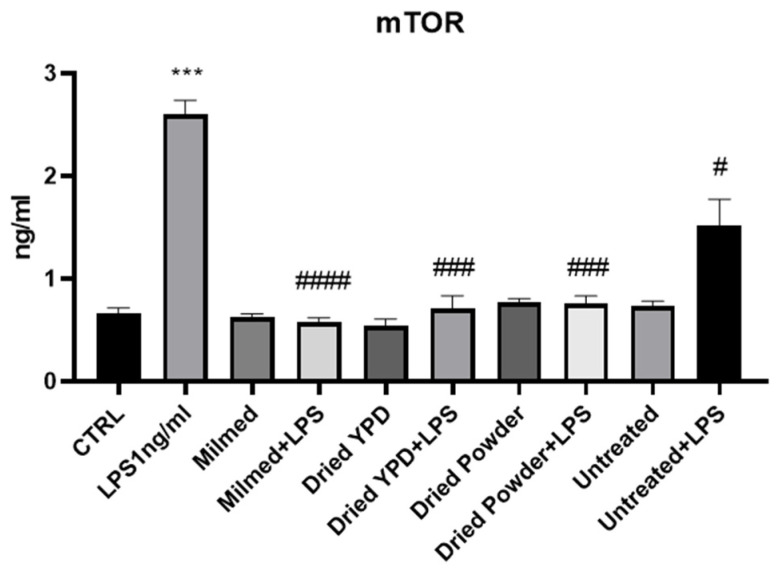
mTOR: a major repressor of autophagic flux. ELISA assay of mTOR was evaluated in BV-2 cells after pre-treatment with Milmed or untreated yeast for 45 min and incubated with LPS 1 ng/mL for 24 h. Data are shown as the mean ± SD from three independent experiments performed in duplicate using the unpaired Student’s *t* test. *** *p* < 0.001, # *p* < 0.05, ### *p* < 0.001, #### *p* < 0.0001 * vs. CTRL; # vs. LPS.

mTOR is a major repressor of autophagic flux. The addition of Milmed as such or in the form of dried powder inhibited mTOR stimulated by the incubation of BV2 microglia with LPS, thereby removing one of the main pathways of inhibition of autophagic flux. Protein expression analysis of mTOR (Figure 3) significantly increased in the presence of LPS while drastically decreasing after 24 h after treatment with modified yeast (Milmed, dried YPD, and dried powder). There was also a slight decrease in the untreated yeast treatment in the presence of LPS. In the absence of LPS, there was no significant difference with the CTRL.

LC3 is a protein involved in autophagy that exists in two main forms: LC3-I, which is present in the cytoplasm, and LC3-II, which is associated with the membranes of autophagosomes. In response to stress signals, such as amino acid deficiency, autophagy is activated, increasing the conversion of LC3-I to LC3-II. This process indicates the formation of autophagosomes, making LC3-II a reliable marker for monitoring autophagy activity [45]. A commonly accepted signal for the induction or activation of autophagy refers to increased levels of LC3-II or decreased levels of p62, an autophagic substrate that is degraded through the autophagic process [46].

**Figure 4:**
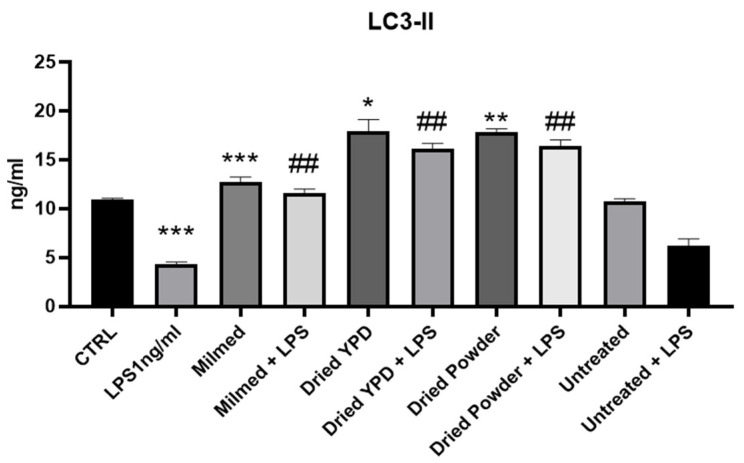
LC3-II is a reliable marker for monitoring autophagy activity. ELISA assay of LC3-II was evaluated in BV-2 cells after pre-treatment with Milmed or untreated (control) yeast for 45 min and incubated with LPS 1 ng/mL for 24 h. Data are shown as the mean ± SD from three independent experiments performed in duplicate using the unpaired Student’s *t* test. * *p* < 0.05, ** *p* < 0.01, *** *p* < 0.001, ## *p* < 0.01 * vs. CTRL; # vs. LPS.

In Figure 4, it can be seen that LPS treatment significantly decreased the LC3-II protein expression while modified yeast significantly increased the LC3-II protein expression compared with both the untreated BV2 cells (CTRL) and BV2 cells treated only with LPS. Untreated yeast added to cultures did not modulate LC3-II expression in the presence or in the absence of LPS.

### 3.2. Antioxidant Effect of Milmed in BV2 Cells

Nrf2 is a transcription factor present in all cells that is activated by oxidative stress. It binds to the antioxidant response element (ARE), an enhancer sequence found in the regulatory regions of antioxidant genes such as *SOD1* and *GPX* [47].

**Figure 5:**
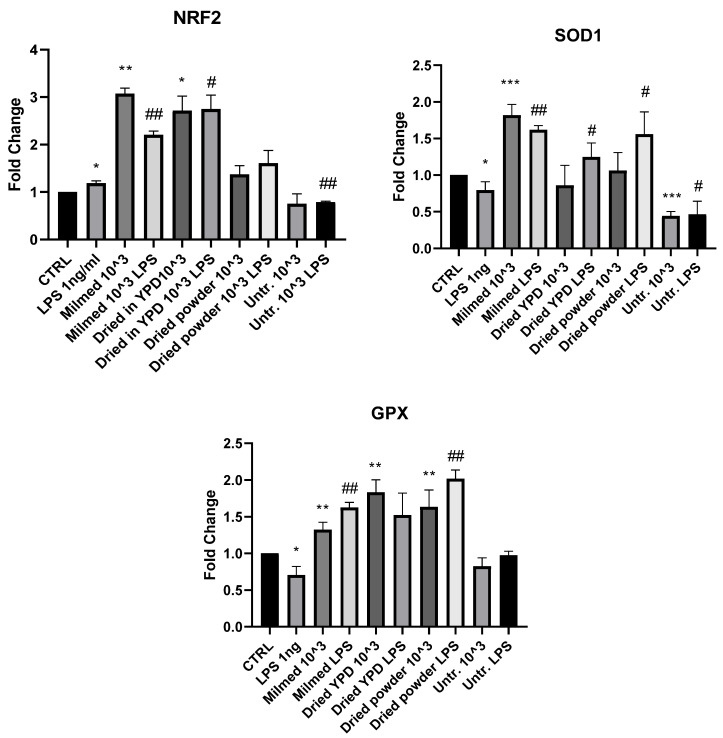
mRNA expression of *Nrf2, SOD1*, and *GPX* in BV-2 cells after yeast pre-treatment and LPS stimulation. mRNA expressions of *Nrf2, SOD1*, and *GPX* were evaluated in BV-2 cells after pre-treatment with Milmed or untreated yeast for 45 min and incubated with LPS 1 ng/mL for 4 h by qRT-PCR. Data are shown as the mean ± SD from three independent experiments performed in triplicate. Expression profiles were determined using the 2^−ΔΔCT^ method. vs. CTRL * *p* < 0.05, ** *p* < 0.01, *** *p* < 0.001; vs. LPS # *p* < 0.05, ## *p* < 0.01.

The analysis of *Nrf2* mRNA expression 4 h after pretreatment with Milmed yeast in the presence and absence of LPS demonstrated that both liquid Milmed and the dried form regrown in YPD significantly increased *Nrf2* expression in both the presence and absence of the inflammatory LPS stimuli. Powder directly added to the culture did not modulate *Nrf2* expression. Pre-treatment with untreated yeast decreased *Nrf2* mRNA expression in the presence of LPS compared with BV2 cells treated with LPS alone. Treatment with LPS significantly reduced the mRNA expression of *SOD1* and *GPX.* After 4 h after pretreatment, as shown in Figure 5, there was an increase in the mRNA expression of *SOD1* and *GPX* in the liquid Milmed conditions both in the presence and absence of LPS. *SOD1* mRNA expression increased significantly in BV2 cells treated with dried YPD and dried powder in the presence of LPS while *GPX* mRNA expression increased significantly in cells treated with dried YPD and dried powder compared with the CTRL group. In the presence of LPS, an increase in *GPX* mRNA expression was observed to be significant with the addition of dried powder. Treatment with untreated yeast significantly decreased *SOD1* mRNA expression both in the presence and absence of LPS. The same treatment did not appear to modulate *GPX* expression.

**Figure 6:**
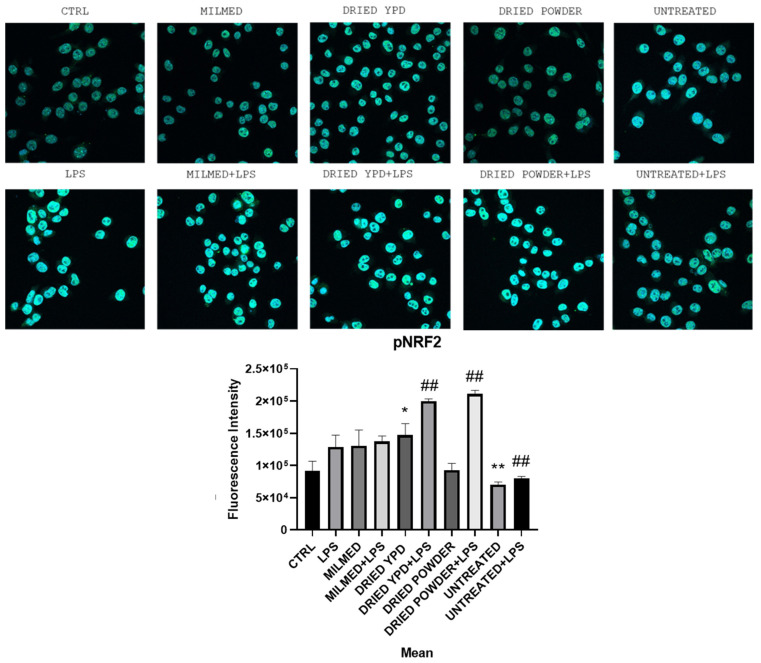
The bar graph shows the fluorescence intensity related to pNRF2 expression under different experimental conditions. Data are shown as the mean ± SD from three independent experiments (24 h). Magnification 63×. vs. LPS ## *p* < 0.01; vs. CTRL * *p* < 0.05, ** *p* < 0.01.

Analysis of the active form of NRF2, phosphorylated NRF2 (pNRF2), was evaluated by immunuofluorescence, and in Figure 6, it is possible to observe that only a few conditions showed more pronounced changes on pNRF2 expression. In particular, two conditions showed a significant increase compared with the others, which were dried grown in YPD compared with CTRL. Both the dried YPD form and dried powder in the presence of LPS significantly increased the expression of pNRF2. The dried powder condition showed a reduction compared with CTRL, although this was not found to be significant.

### 3.3. Milmed Yeast Extends Lifespan and Modulates Growth in C. elegans

**Figure 7:**
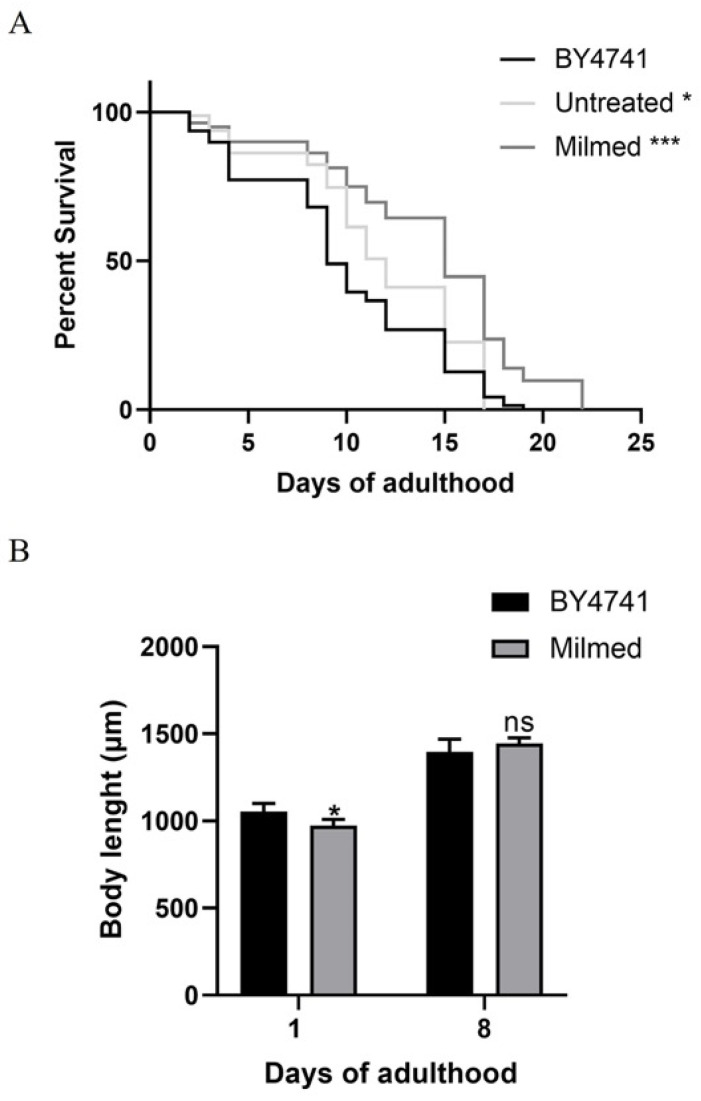
Effect of Milmed yeast on nematode lifespan and body length. (**A**) Kaplan–Meier survival plot of N2 worms fed with Milmed or untreated yeast compared with the *S. cerevisiae* BY4741-fed worms used as controls. *n* = 80 per data point in individual experiments. (**B**) Effect of yeast feeding on the body size of *C. elegans*. Worms were grown in the presence of Milmed yeast, untreated yeast, or *S. cerevisiae* BY4741 (control), and their body lengths were measured from head to tail at the indicated time points. Bars represent the mean of three independent experiments. Asterisks indicate statistically significant differences (* *p* < 0.05, *** *p* < 0.001); ns: not significant.

To investigate the effects of yeast on *C. elegans* physiology, we first examined the survival rate (Figure 7A). The median lifespan of wild-type nematodes fed with Milmed yeast from embryo hatching was significantly extended compared with the *S. cerevisiae* BY4741-fed controls. Indeed, the 50% survival rate in the Milmed-fed worms was recorded at day 13, whereas it was observed at day 11 and day 9 in the untreated and BY4741-fed animals, respectively. Microscopic analysis of larval development revealed that the Milmed diet slightly affected the body size (Figure 7B). Body length analysis showed a slight reduction at day 1 of adulthood, but progressively increased with age in both conditions. However, at later stages, the body length of the Milmed-fed worms became comparable to that of the control group.

### 3.4. Milmed Yeast Supplementation Reduces Age-Related Oxidative Stress in C. elegans

**Figure 8:**
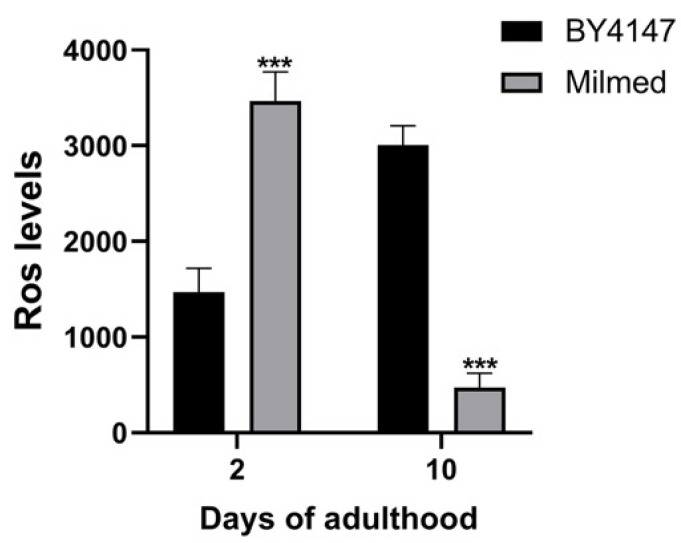
Measurement of ROS levels in Milmed-fed worms at 2 and 10 days of adulthood. Worms fed *S. cerevisiae* BY4741 were taken as the control. Statistical analysis was evaluated by one-way ANOVA with the Bonferroni post-test; asterisks indicate significant differences (*** *p* < 0.001). Bars represent the mean of three independent experiments.

Since excessive oxidative damage can impair cellular function and has been shown to increase with age [48], and given that reactive oxygen species (ROS) are one of the primary causes of aging, the ROS levels were analyzed in *C. elegans* following yeast-based dietary supplementation (Figure 8). These ROS are known to induce oxidative damage to essential cellular components including DNA, proteins, and lipids [41]. At day 2 of adulthood, the ROS levels in Milmed-fed worms were approximately twice as high as in the control group (Figure 8). However, by day 10 of adulthood, while the ROS levels in control worms had doubled compared with the young adults—as typically observed during aging—the Milmed-fed nematodes exhibited an over 80% reduction in ROS levels. This suggests that the Milmed diet may enhance cellular antioxidant defenses, thereby mitigating age-related oxidative stress and contributing to the observed lifespan extension.

In *C. elegans*, the oxidative stress response is primarily controlled by the SKN-1/Nrf2 transcription factor, which plays a key role in maintaining redox balance by regulating the expression of various antioxidant genes. The *gst-4* (glutathione S-transferase) gene encodes a phase II detoxification enzyme that is strongly induced in a SKN-1-dependent manner [42].

**Figure 9:**
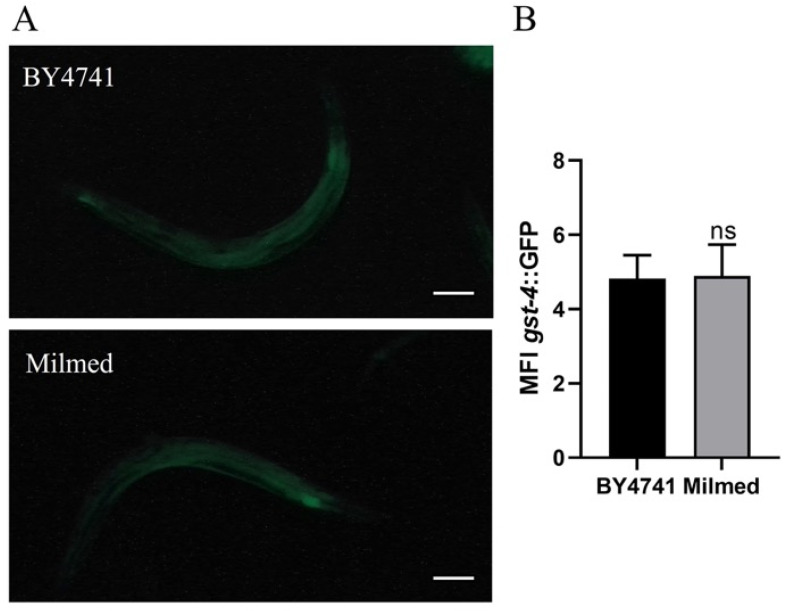
Fluorescence microscopy of *gst-4*::GFP transgenic strain. (**A**) Fluorescence microscopy of *gst-4*::GFP worm strain fed with Milmed from embryo hatching and (**B**) related MFI. Scale bar = 100 μm. Untreated yeast-fed worms were taken as the control. Statistical analysis was evaluated by one-way ANOVA with the Bonferroni post-test; ns: not significant. Bars represent the mean of three independent experiments.

In this study, we utilized transgenic *C. elegans* expressing the *gst-4::GFP* reporter to assess potential differences in the expression of this detoxifying enzyme (Figure 9). However, during aging, no significant variations were observed, as further confirmed by the mean fluorescence intensity (MFI) analysis.

### 3.5. Milmed Yeast Extends Lifespan in C. elegans Through Activation of SKN-1/Nrf2

**Figure 10:**
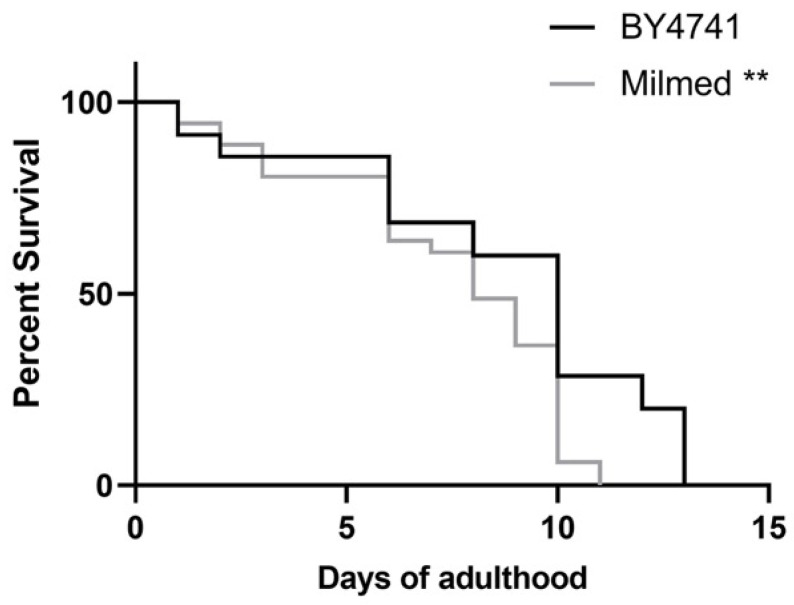
Effect of Milmed on *skn-1* mutant animals. Kaplan–Meier survival plot of *skn-1* mutant worms fed Milmed yeast from embryo hatching. Lifespans of untreated-fed worms (control) were taken as the reference; *n* = 80 for each data point of the single experiments (** *p* < 0.01). The experiment was performed in triplicate.

On the other hand, the viability was further examined by administering Milmed yeast to worms carrying mutations in the *skn-1* gene, which encodes the transcription factor involved in the p38 MAPK pathway (Figure 10). The median lifespan of *skn-1* mutant nematodes treated with Milmed from embryo hatching was not only deprived of the pro-longevity effect, but was also significantly reduced (Figure 10). In particular, the 50% survival rate in Milmed-fed *skn-1* mutants was recorded at day 8 compared with day 9 in the control worms. Thus, Milmed did not induce a pro-longevity effect in these mutants as observed in the wild-type animals, demonstrating that the beneficial effects of Milmed are mediated through the activation of the transcription factor SKN-1/Nrf2.

## 4. Discussion

As outlined above, modulation of microglia activity has recently been proposed as a therapeutic strategy to slow down the progression of neurodegenerative diseases. Gut microbiota has been shown to play an important role in the modulation of neuroinflammation and neurodegeneration; moreover, dysbiosis has been related to neurodegenerative acceleration. Our previous results showed that Milmed yeast, obtained from *S. cerevisiae*, after exposure to electromagnetic millimeter wavelenghts, induced a reversal of LPS-M1 polarized microglia toward an anti-inflammatory phenotype as demonstrated by the decrease in the mRNAs of IL-1β, IL-6, and TNF-α and in the expression of iNOS as well as by the increase in IL-10 mRNA and the expression of arginase-1 [27]. Recently, the importance of disabled macro-autophagy in aging and in the development of neurodegenerative diseases, including AD and PD, has been highlighted [49]. Several findings showed a direct correlation between dysbiosis, neuroinflammation, and the development of several neurodegenerative diseases including AD, PD, dementia, and depression [50]. Furthermore, a direct correlation has been hypothesized between aging and mitochondrial dysfunction, cellular senescence, disabled macro-autophagy, chronic inflammation, and dysbiosis [51]. Non-communicable diseases (NCDs) represent 77% of all deaths in Europe and remain the most widespread and without effective therapy; many of them share pathogenetic mechanisms and show a high degree of molecular connectivity. The study of these interconnections occurs through the identification of one or more mechanisms underlying the disease and its comorbidities. This approach, thanks to the regulation of a common molecular target can provide, at least partially, a therapeutic benefit to regulate different altered cellular responses. These alterations constitute a mosaic of significantly unbalanced processes that end up influencing each other, disrupting mechanisms that should guarantee the homeostasis of the cells. Therefore, an interdependence between these different alterations is created, whereby the accentuation or attenuation of one specific hallmark usually also affects other hallmarks.

The onset and development of several neurodegenerative diseases, including Alzheimer’s disease, Parkinson’s disease, schizophrenia and depression, have been related to neuroinflammation [52]. Neuroinflammation is characterized by the polarization of microglial cells toward a pro-inflammatory phenotype, M1 or classically activated phenotype, which releases inflammatory cytokines and chemokines, showing a decreased phagocytic capability and an increased chemotactic activity. The process is induced and amplified by oxidative stress, targeting the transcription factors that bind to the promoter regions of inflammatory genes. For all these reasons, the modulation of microglia activity has recently been proposed as a therapeutic strategy to slow down the progression of neurodegenerative diseases [53].

Our present study focused on Milmed actions that promote autophagy in microglia cells exposed to an inflammatory microenvironment and to promote longevity, as shown in the *C. elegans* experimental model. Disabled macro-autophagy accelerates the aging process, whereas, in contrast, autophagy is a major anti-aging mechanism involved in pro-longevity pathways [54]. In a recent review published in 2023, the importance of disabled macro-autophagy, chronic inflammation, and dysbiosis in accelerating the aging process was highlighted. The interdependence of aging hallmarks implies that the experimental accentuation or attenuation of one specific hallmark usually affects other hallmarks concurrently. Our findings demonstrate that Milmed exerted significant pro-longevity effects in *C. elegans*, extending the lifespan and improving stress resistance. The rise in ROS levels observed on the first day of adulthood seems to indicate a positive effect rather than a harmful one and appears to trigger oxidative defense systems, which help lower the ROS levels during aging and may contribute to a longer lifespan This was further confirmed by previous studies highlighting the role of probiotics, including yeast-based supplements, in modulating oxidative stress and inflammation [55,56]. The present results align with these findings and suggest that Milmed yeast may function as a bioactive dietary component capable of enhancing cellular stress responses. Notably, the ability of Milmed to lower ROS accumulation in aging worms suggests a potential protective mechanism against age-related oxidative damage, which is a hallmark of cellular aging and neurodegenerative diseases [57]. The observed reduction in ROS levels (Figure 8) among the aged worms and the loss of the pro-longevity effect in *skn-1* mutants (Figure 10) indicated that the p38 MAPK signaling cascade plays a crucial role in mediating these anti-aging effects. This contention is further supported by previous studies highlighting the role of probiotics, including yeast-based supplements, in modulating oxidative stress and inflammation [55,56]. The present results conform with these findings and suggest that Milmed yeast may function as a bioactive dietary component capable of enhancing cellular anti-stress responses. Notably, the ability of Milmed to lower ROS accumulation in aging worms suggests a potential protective mechanism against age-related oxidative damage, which is a hallmark of cellular aging and neurodegenerative diseases [57].

Autophagy also plays a key role in extending longevity. A proteostasis (protein homeostasis) imbalance may result from the dysregulation of autophagy, a well-known clearance mechanism of aggregated proteins, due to changes in mTOR/AMPK activity [58,59]. Indeed, mTOR inhibition with rapamycin reduces toxicity in animal models of NDs [60,61]. Additionally, given that autophagy has a central role in the removal of defective mitochondria, its regulation by mTOR/AMPK may also be associated with mitochondrial homeostasis and respiration [62,63]. In a previous study, *S. cerevisiae*-derived vacuoles added to LPS-treated human neuroblastoma cells reduced phosphorylated tau and β-amyloid expression and confirmed our previous data showing that Milmed decreased the expression of pro-inflammatory cytokines as well as iNOS. Furthermore, it was shown that *S. cerevisiae*-derived vacuoles regulated NF-kBp65 translocation to the nucleus [64]. The administration of *Saccharomyces boulardi* to Drosophila that were exposed to paraquat, a mitochondrial toxin, resulted in improved longevity and motor function. The main activity resulted in increased lysosomal degradation of dysfunctional mitochondria, which is mitophagy, outlining the potential therapeutic benefit of probiotic administration to patients with Parkinson’s disease [65]. In recent years, it has been shown that the autophagy machinery is used for cellular activities independent from lysosomal degradation, opening a new chapter in understanding the broad spectrum of functions of autophagy, outlining the pro-survival role of autophagy [66,67].

It has been evidenced repeatedly that oxidative stress presents one of the main factors promoting chronic degenerative diseases, and several environmental conditions favoring the increase in ROS inside cells have been included among the main risk factors for the development of cardiovascular and neurodegenerative diseases; therefore it is of fundamental importance for pro-longevity interventions to contain the cellular concentrations of ROS within the physiological limits [68]. Our study demonstrated that Milmed is able to enhance the antioxidant defenses of the cell, as demonstrated by the modulation of the expression of NRF2, SOD3, and GPX, even in the presence of LPS and the translocation of phosphorilated-NRF2 to the nucleus of the cell. The in vivo *C. elegans* model confirmed the antioxidant effects of Milmed in the reduction of ROS. Concomitantly, the antioxidant potential of some strains of *S. cerevisiae* has also been demonstrated in other studies [69]. For example, Siesto et al. showed the antioxidant potential of *Saccharomyces cerevisiae* used in wine fermentation and correlated this activity to the content of beta-glucans [70]. Yeast β-glucan has various biological properties including immunomodulatory, anti-inflammatory, and antioxidant [71]. As a matter of fact, our study confirms the activity of Milmed as a powerful antioxidant.

## 5. Conclusions

Milmed yeast demonstrated a notable and marked pro-autophagic and antioxidant activity which, combined with the previously demonstrated anti-inflammatory activity, highlights its role as a highly effective probiotic for its beneficial effects on health and longevity.

## Figures and Tables

**Table 1:** List of primers.

GENE	Forward Primer (5′–3′)	Reverse Primer (5′–3′)	Accession Numbers	bp
mLC3	TTCTTCCTCCTGGTGAATGG	GTCTCCTGCGAGGCATAAAC	NM_026160	2455
mBeclin-1	CAGCCTCTGAAACTGGACACGA	CTCTCCTGAGTTAGCCTCTTCC	NM_019584	2072
mNrf2	TCTGAGCCAGGACTACGACG	GAGGTGGTGGTGGTGTCTCTGC	NM_010902	2347
mp62	CCTTGCCCTACAGCTGAGTC	CCACACTCTCCCCCACATTC	NM_001290769	1916
mATG7	CAATGAGATCTGGGAAGCCATAA	AGGTCAAGAGCAGAAACTTGTTGA	NM_001253717	3872
mβ-Actin	GGCTGTATTCCCCTCCATCG	CCAGTTGGTAACAATGCCATGT	NM_007393.5	1935

## Data Availability

The original contributions presented in this study are included in the article. Further inquiries can be directed to the corresponding author.
